# Determinants of Anemia Among Antenatal Care Attendees in Southwest Ethiopia: A Case‐Control Study

**DOI:** 10.1002/hsr2.71290

**Published:** 2025-10-28

**Authors:** Tewodros Yosef, Asaye Gizachew, Nigusie Shifera

**Affiliations:** ^1^ School of Public Health, College of Medicine and Health Sciences Mizan‐Tepi University Mizan Teferi Ethiopia; ^2^ School of Medicine, Faculty of Health Deakin University, Waurn Ponds Geelong Australia

**Keywords:** anemia, hookworm, iron supplementation, malaria, pregnant women

## Abstract

**Background and Aim:**

Anemia is marked by a reduction in red blood cells or hemoglobin levels, leading to impaired oxygen transport. It presents a significant global public health issue, associated with elevated morbidity and mortality, especially among pregnant women and children. Therefore, this study aimed to evaluate the determinants of anemia among pregnant women attending antenatal care (ANC) at selected public health hospitals in Southwest Ethiopia.

**Methods:**

A case‐control study, conducted from January 1 to February 30, 2023, included 374 pregnant women (91 with anemia and 283 without). Data were collected through interviewer‐administered structured questionnaires. SPSS Version 21 was used for data analysis, employing binary logistic regression to identify factors associated with anemia. The significance level was set at 0.05.

**Results:**

After adjusting for confounding variables, the determinants of anemia among pregnant women were identified as lack of iron supplementation [AOR = 2.86, 95% CI (1.45, 3.85)], consumption of hot drinks after a meal [AOR = 1.63, 95% CI (1.03, 2.76)], history of malaria infection [AOR = 4.34, 95% CI (2.35, 7.72)], hookworm infection [AOR = 2.57, 95% CI (1.48, 4.46)], and short birth interval [AOR = 8.64, 95% CI (4.98, 17.6)].

**Conclusion:**

The main factors contributing to anemia among pregnant women include insufficient iron supplementation, consumption of hot drinks after meals, a history of malaria, hookworm infection, and short birth intervals. To reduce anemia prevalence, it is essential to ensure adequate iron supplementation, educate women on the impact of hot drinks after meals, implement malaria prevention and treatment, address hookworm infections through sanitation and deworming, and promote optimal birth spacing through family planning education.

**Abbreviations**
ANC: antenatal care
AOR: adjusted odds ratio
CI: confidence interval
COR: crudes odds ratio
GA: gestational age
Hgb: hemoglobin
MUAC: mid‐upper arm circumference
WHO: World Health Organization

## 1 Introduction

Anemia during pregnancy remains a significant public health concern globally, particularly in low‐ and middle‐income countries, where it is associated with increased risks of maternal and perinatal morbidity and mortality [1, 2]. Anemia is defined as a decrease in red blood cell concentration or hemoglobin concentration, leading to reduced oxygen transport capacity [2]. The World Health Organization (WHO) estimates that anemia affects over 40% of pregnant women worldwide, with higher prevalence rates observed in sub‐Saharan Africa, including Ethiopia [3]. In Ethiopia, anemia among pregnant women is a major health issue, with studies reporting prevalence rates ranging from 20% to 50% depending on the region [4].

Anemia poses a significant public health and nutritional challenge with implications for health, social well‐being, and economic development [5]. Anemia stands as the most common pregnancy complication, affecting over 50% of pregnant women in developing countries [6, 7]. Particularly severe in South East Asia (48.7%) and Africa (46.3%) [2, 8], anemia contributes to ~20% of maternal deaths, with around 510,000 maternal deaths annually linked to childbirth or early postpartum [9]. In sub‐Saharan Africa, the pooled prevalence of anemia among pregnant women is 35.6% [8], with Ethiopia having a 32% prevalence, primarily attributed to factors like poor nutrition, infections, menstrual blood loss, and frequent pregnancies [10].

The etiology of anemia in pregnancy is multifactorial, involving sociodemographic factors, nutritional deficiencies (especially iron, folate, and vitamin B12), infectious diseases such as malaria and helminthiasis, reproductive factors, chronic conditions like HIV/AIDS, and hygiene and sanitation factors [2, 11, 12, 13, 14, 15, 16, 17, 18]. In the context of antenatal care (ANC), identifying and addressing the determinants of anemia is crucial for implementing effective interventions aimed at improving maternal and fetal outcomes. However, there is limited evidence on the specific determinants of anemia among ANC attendees in Southwest Ethiopia, a region characterized by diverse sociodemographic and cultural factors that may influence maternal health.

Despite multiple efforts made by the government and other stakeholders, anemia during pregnancy is still a public health problem in Ethiopia [19] including the study area. Therefore, this study aimed to evaluate the determinants of anemia among pregnant women attending ANC at selected public health hospitals in Southwest Ethiopia.

## 2 Methods

### 2.1 Study Design, Setting, and Period

An unmatched case‐control study was conducted among ANC attending pregnant women at selected public health hospitals (Mizan‐Tepi University Teaching Hospital, Gebretsadik Shewa General Hospital, and Bachuma Primary Hospital) in Southwest Ethiopia. Mizan‐Tepi University Teaching Hospital, located in Ethiopia's southwest Bench Sheko Zone, serves communities in Bench Sheko, West Omo, Sheka, and Gambela regions. Established in 1986 as Mizan Teferi Hospital and integrated into Mizan‐Tepi University in 2016, it is situated 580 km southwest of Addis Ababa. Gebretsadik Shewa General Hospital, situated in Bonga town, Kaffa Zone, is 449 km from Addis Ababa. Bachuma Primary Hospital, located in the West Omo Zone, was upgraded from a health center in 2017 and is ~660 km from Addis Ababa and 180 km from Bonga town. The study was conducted from January 1 to February 30, 2023.

### 2.2 Populations

The study focused on pregnant women attending selected public health hospitals for ANC. The participants were divided into two groups: cases, consisting of pregnant women with anemia (as defined by their hemoglobin levels), and controls, consisting of those without anemia. All pregnant women attending ANC services during the study period were eligible for inclusion. Women who were too ill, did not consent, had chronic illnesses, or had received blood transfusions in the past 3 months were excluded.

### 2.3 Sample Size Determination and Sampling Technique

The sample size was determined using Epi Info Version 7.2 software, based on the following assumptions: the expected proportion of chronic illness among anemic mothers was estimated at 17%, while among nonanemic mothers, it was estimated at 5.7% [18], 95% confidence level, 80% power, and a ratio of 1:3. The initial sample size was 340. Thus, after considering the 10% nonresponse rate, the final sample size was 374 (91 cases and 283 controls). Sample allocation to the three hospitals was determined based on the proportion of women attending ANC services at each facility. Cases were sampled consecutively until the desired number was achieved, with three controls selected for each case on the same day using a consecutive sampling method.

### 2.4 Data Collection Tools, Procedures, and Quality Control

Data were collected through a combination of questionnaires, laboratory tests (hemoglobin measurement and stool examination), and anthropometric measurements. The questionnaire, initially in English and translated into Amharic, was used in face‐to‐face interviews to gather information on sociodemographic, hygiene, nutrition, and health factors, as well as obstetrics and gynecology history. Nutritional status was assessed using middle upper arm circumference (MUAC), and hemoglobin levels were measured using HemoCue photometers. Stool examinations were conducted to detect intestinal hookworm parasites. Stool samples were collected using clean, leakproof cups and examined microscopically within 30 min. A wet mount prepared with saline and/or iodine was used to identify intestinal hookworm parasites. The data collection was handled by trained three medical laboratory technicians and three midwifery professionals.

### 2.5 Study Variables and Operational Definitions

The dependent variable was anemia. The independent variables included sociodemographic and economic factors (age, residence, education, marital status, occupation), gynecological and obstetric factors (gestational age, gravidity, parity, history of abortion, number of children, birth interval), and hygiene, disease, and nutrition‐related factors (nutritional status, hookworm infections, malaria history, iron supplementation, consumption of hot drinks after meals, and intake of raw vegetables).

Gestational age was classified based on the last normal menstrual period: < 14 weeks for the first trimester, 14–27 weeks for the second trimester, and above 28 weeks for the third trimester [20]. Anemia was defined as hemoglobin levels of ≤ 11 g/dL in the first and third trimesters, and < 10.5 g/dL in the second trimester [20], otherwise nonanemic. Nutritional status was assessed using MUAC, with ≥ 23 cm considered normal and < 22 cm indicating undernutrition [21, 22]. Iron supplementation involved daily intake of ferrous sulfate, fumarate, or gluconate for at least 90 days [10].

Abortion is the spontaneous or induced termination of a pregnancy before the full 28 weeks of gestational age or before the viability of the fetus [4]. A short birth interval was defined as < 33 months from the previous live birth, while an optimal birth interval was 33 months or more [23].

### 2.6 Data Processing and Analysis

Data were analyzed using Statistical Package for the Social Sciences (SPSS) version 21.0 (IBM Corp. Released 2012. IBM SPSS Statistics for Windows: Armonk, NY: IBM Corp.). The outcome variable was binary (0: nonanemic, 1: anemic). Binary logistic regression assessed the relationship between independent variables and the outcome. Variables with *p* < 0.25 [24] in bivariable analysis were included in multivariable logistic regression. The Hosmer–Lemeshow test (*p* = 0.435) indicated a well‐fitting model, with no significant interactions found. Multivariable analysis identified factors significantly associated with anemia, using adjusted odds ratios (AOR) with 95% confidence intervals and *p* values < 0.05 to confirm significance.

## 3 Results

### 3.1 Sociodemographic and Reproductive Factors

A total of 355 pregnant women participated in the study, with 273 controls and 82 cases, yielding response rates of 96.5% for controls and 90.1% for cases. Most participants were under 30 years old, with a higher proportion in the control group (78.4%) compared to the case group (75.6%). Rural residents comprised 40.2% of the case group and 50.6% of the control group. Most participants were married, with 93.9% in the case group and 96.7% in the control group. Government employees were more prevalent in the control group (26.7%) than in the case group (20.7%). A history of unsafe abortion was reported by 30.5% of the case group and 8.1% of the control group. Additionally, 48.8% of the case group and 8.8% of the control group had experienced a short birth interval (Table 1).

**Table 1:** Sociodemographic and reproductive factors of ANC attendees in Southwest Ethiopia.

Variables	Categories	Cases	Controls	*χ* ^2^ *p*
Age	< 30	62 (75.6)	214 (78.4)	0.352
	≥ 30	20 (24.4)	59 (21.6)	
Residence	Rural	33 (40.2)	138 (50.6)	0.049
	Urban	49 (59.8)	135 (49.4)	
Marital status	Single/separated	5 (6.1)	9 (3.3)	0.673
	Married	77 (93.9)	264 (96.7)	
Education	Unable to read and write	28 (34.2)	116 (42.5)	0.245
	Primary school	24 (29.3)	74 (27.1)	
	Secondary school and above	30 (37.5)	83 (30.4)	
Occupation	Government employee	17 (20.7)	73 (26.7)	0.825
	Others^a^	65 (79.3)	200 (73.3)	
Unsafe abortion	Yes	25 (30.5)	22 (8.1)	0.025
	No	57 (69.5)	251 (91.9)	
Short birth interval	Yes	40 (48.8)	24 (8.8)	0.002
	No	42 (51.2)	249 (91.2)	

^a^ Others: housewife, merchant, farmer, daily laborers, and students.

### 3.2 Hygiene, Nutritional, and Disease Related Factors

**Figure 1:**
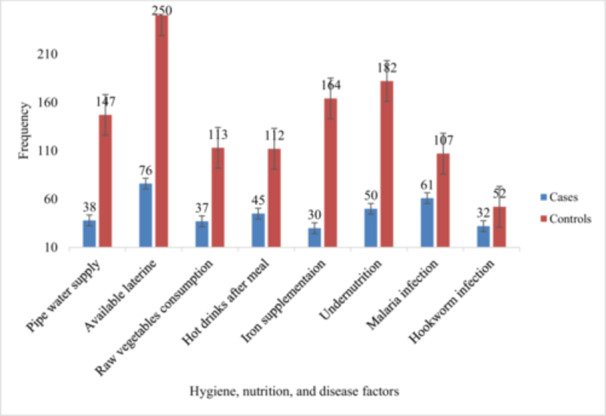
Health, nutrition, and disease factors among ANC attendees in Southwest Ethiopia.

In total, 38 (46.3%) of cases and 147 (53.9%) of controls used pipe water; 61 (74.4%) of cases and 107 (39.2%) of controls had a history of malaria infection; 32 (39%) of cases and 52 (19.1%) of controls had hookworm infection. More cases (54.9%) reported consuming hot drinks after meals compared to controls (41%) (Figure 1).

### 3.3 Determinants of Anemia

After adjusting for confounding variables, the determinants of anemia among pregnant women were identified as lack of iron supplementation [AOR = 2.86, 95% CI (1.45, 3.85)], consumption of hot drinks after a meal [AOR = 1.63, 95% CI (1.03, 2.76)], history of malaria infection [AOR = 4.34, 95% CI (2.35, 7.72)], hookworm infection [AOR = 2.57, 95% CI (1.48, 4.46)], and short birth interval [AOR = 8.64, 95% CI (4.98, 17.6)] (Table 2).

**Table 2:** Determinants of anemia among ANC attendees in Southwest Ethiopia.

Variables	Categories	Cases	Controls	COR, 95% CI	AOR, 95% CI
Iron supplementation	Yes	30 (36.6)	164 (60.1)	1	1
	No	52 (63.4)	109 (39.9)	2.63 (1.56, 4.35)**	2.86 (1.45, 3.85)**
Undernutrition	Yes	50 (61)	182 (66.7)	0.78 (0.47, 1.30)*	0.74 (0.45, 1.34)
	No	32 (39)	91 (33.3)	1	1
Unsafe abortion	Yes	25 (30.5)	22 (8.1)	5.00 (2.64, 9.50)**	4.65 (0.96, 9.22)
	No	57 (69.5)	251 (91.9)	1	1
Short birth interval	Yes	40 (48.8)	24 (8.8)	9.88 (5.41, 18.05)**	8.64 (4.98, 17.6)**
	No	42 (51.2)	249 (91.2)	1	1
Hookworm infection	Yes	32 (39)	52 (19.1)	2.72 (1.59, 4.65)**	2.57 (1.48, 4.46)**
	No	50 (61)	221 (80.9)	1	1
Malaria history	Yes	61 (74.4)	107 (39.2)	4.51 (2.59, 7.83)**	4.34 (2.35, 7.72)**
	No	21 (25.6)	166 (60.8)	1	1
Hot drinks after meal	Yes	45 (54.9)	112 (41)	1.75 (1.06, 2.87)**	1.63 (1.03, 2.76)**
	No	37 (45.1)	161 (59)	1	1

*Note:* 1 = reference value.

Abbreviations: AOR, adjusted odds ratio; CI: confidence interval; COR, crude odds ratio.

* *p* < 0.25;

** *p* < 0.05.

## 4 Discussion

Anemia during pregnancy remains a major public health issue, especially in low‐ and middle‐income countries [1]. This study, conducted among pregnant women attending ANC in Southwest Ethiopia, identified key determinants of anemia, including lack of iron supplementation, consumption of hot drinks after meals, history of malaria and hookworm infections, and short birth intervals.

Iron supplementation was significantly associated with anemia in pregnant women. The odds of anemia among pregnant women with lack of iron supplementation were 2.9 times higher than among pregnant women who had iron supplementation. This study was consistent with studies conducted elsewhere [25, 26]. Iron supplementation during pregnancy has been found to lower the prevalence of anemia and enhance pregnancy outcomes [27]. According to a systematic review by Peña‐Rosas et al. [28], daily oral iron supplementation significantly reduces the risk of maternal anemia, iron deficiency, and low birth weight.

The consumption of hot drinks immediately after meals was significantly associated with an increased risk of anemia in pregnant women. Pregnant women who regularly consumed hot drinks right after eating had a 63% higher likelihood of developing anemia compared to those who did not engage in this practice. This finding was further supported by studies conducted in other regions [29]. This could be because hot beverages, particularly tea and coffee, contain compounds like tannins and polyphenols, which can interfere with the absorption of essential nutrients such as iron [30]. When hot drinks are consumed right after a meal, they can bind to iron in the digestive tract, reducing its bioavailability and preventing the body from absorbing enough iron. Over time, this interference with iron absorption can lead to iron deficiency, a common cause of anemia [31].

Pregnant women with malaria had a 4.3 times higher likelihood of developing anemia compared to those without the infection. The findings of this study align with previous research [26, 32, 33, 34], which consistently reported a strong link between malaria and anemia in pregnant women. This association is likely due to the impact of malaria on the body's ability to maintain healthy red blood cell levels, as the infection leads to hemolysis (destruction of red blood cells) and disrupts the body's ability to produce new red blood cells. There is increased destruction and removal of infected and uninfected erythrocytes, decreased erythrocyte production, and suppression of the erythropoietin response, all of which cause malarial anemia [35, 36].

Hookworm infection was strongly linked to anemia in pregnant women, with those infected being 2.6 times more likely to develop anemia compared to those without the infection. The findings of this study are consistent with several studies conducted in Ethiopia [17, 26, 29, 32, 33, 37], which also reported a similar link between hookworm infection and increased risk of anemia in pregnant women. Intestinal parasites, such as hookworms, contribute to anemia by causing blood loss, nutrient depletion (iron, folic acid, and vitamin B12), and reduced appetite. They also impair digestion by damaging the intestinal lining and interfering with nutrient absorption [38].

Pregnant women with a short birth interval face significantly higher risks of developing anemia, with the odds of anemia being 8.6 times greater compared to those with an optimal birth interval. The findings from this study are consistent with research conducted in Harar, Ethiopia [39, 40], which also showed a strong association between short birth intervals and an increased risk of anemia in pregnant women. A short birth interval is linked to adverse maternal outcomes like anemia, as it does not allow enough time for the mother to recover from the nutritional and physical demands of the previous pregnancy [41]. The increased need for nutrients like iron, folic acid, and calcium during pregnancy, combined with insufficient recovery time, makes the mother more prone to deficiencies and anemia in the next pregnancy. This can result in lower maternal reserves of essential nutrients, further increasing the risk of anemia and other complications [42].

### 4.1 Strengths and Limitations

This hospital‐based unmatched case‐control study effectively identified key determinants of anemia among pregnant women through comprehensive data collection, including interviews, lab tests, and anthropometric measurements. A small sample size can result in wide confidence intervals, reduced power, increased variability, and potential bias, which may limit the reliability and generalizability of the findings. Seasonal variations in malaria and hookworm infection rates may affect the applicability of results across different times of the year. The study did not account for other potential factors like genetic disorders and was limited in generalizability due to its hospital‐based design, focusing only on ANC attendees.

## 5 Conclusion and Recommendations

The main factors contributing to anemia among pregnant women include insufficient iron supplementation, consumption of hot drinks after meals, a history of malaria, hookworm infection, and short birth intervals. To reduce anemia prevalence, it is essential to ensure adequate iron supplementation, educate women on the impact of hot drinks after meals, implement malaria prevention and treatment, address hookworm infections through sanitation and deworming, and promote optimal birth spacing through family planning education.

## Author Contributions

**Tewodros Yosef:** conceptualization, investigation, writing – original draft, writing – review and editing, methodology, validation, formal analysis, data curation, software, resources, visualization. **Asaye Gizachew:** investigation, data curation, funding acquisition, formal analysis, project administration, writing – review and editing, supervision. **Nigusie Shifera:** funding acquisition, project administration, formal analysis, supervision, writing – review and editing, investigation, data curation. All authors have read and approved the final version of the manuscript.

## Ethics Statement

Ethical clearance for the study was granted by the Research Review Committee of Mizan‐Tepi University, College of Medicine and Health Sciences (PGC/115/2022).

## Consent

Written informed consent was obtained from each participant. Participants were assured of the confidentiality of their data and informed about the study's objectives, their right to withdraw, and the protection of their personal information.

## Conflicts of Interest

The authors declare no conflicts of interest.

## Transparency Statement

The lead author, Tewodros Yosef, affirms that this manuscript is an honest, accurate, and transparent account of the study being reported; that no important aspects of the study have been omitted; and that any discrepancies from the study as planned (and, if relevant, registered) have been explained.

## Data Availability

The data supporting this study's findings are available from the corresponding author on reasonable request. T.Y. had full access to all of the data in this study and takes complete responsibility for the integrity of the data and the accuracy of the data analysis.
